# Overexpression of RACK1 Predicts Poor Prognosis in Melanoma

**DOI:** 10.7150/jca.36905

**Published:** 2020-01-01

**Authors:** Congcong Shen, Hui Hua, Lixiong Gu, Shuanglin Cao, Hengji Cai, Xiaodong Yao, Xiaodong Chen

**Affiliations:** 1 Department of Dermatology, Affiliated Hospital of Nantong University, Nantong, 226001, P.R. China; 2 Department of Dermatology, The Third People's Hospital of Nantong, Nantong, 226001, P.R. China

**Keywords:** RACK1, melanoma, prognosis, apoptosis

## Abstract

Melanoma is a highly malignant skin cancer with limited treatment options, the mechanism of the occurrence and development of melanoma is still unclear till now. Receptor for activated C kinase 1 (RACK1) is a scaffolding protein that mediates multiple signaling pathways; it interconnects distinct signaling pathways to control essential cellular processes. RACK1 was reported as an oncogene in human tumorigenesis, but little is known about its role in melanoma. This study aimed to investigate the expression of RACK1 in patients with melanoma and to reveal its possible functions in melanoma cells. The expression profiles of RACK1 detected in tumor tissues from melanoma patients showed that RACK1 was higher in tumor tissues, and its expression level was well associated with the clinical progression of melanoma (TNM stage, P=0.009). Furthermore, RNA interfering (RNAi) knockdown of RACK1 could efficiently suppress the proliferation, migration and invasion of A375 and A875 cells and promote their apoptosis. Taken together, these results suggest that RACK1 may be a poor prognostic factor in human melanoma, and it may be a new therapeutic target for melanoma treatment.

## Introduction

Melanoma is a malignant skin cancer with aggressive forms, originating from pigment-containing cells known as melanocytes in the basal layer of the epidermis, being responsible for high mortality of skin cancers 1, 2. The main cause of melanoma is due to the DNA damage resulting from exposure to the sun or other sources of ultraviolet light in people with low skin pigmentation, genetic factors also plays a role in melanoma occurrence. In recent years, many studies have been reported on the occurrence and development of melanoma, but its molecular pathological mechanism is still unclear 3-5. In current, due to the high malignancy of melanoma, there are few treatment options. Although target therapy strategies have been widely used in the treatment of many human cancers, its application in melanoma still has many limitations, many patients with advanced melanoma cannot achieve tumor regression 6, 7. Therefore, it is urgent to clarify the molecular mechanism of melanoma development and to develop novel gene targets for its clinical diagnosis and therapy 8.

Receptor for activated C kinase 1 (RACK1) was first cloned from the cDNA library of a chicken genomic DNA clusters and human B-lymphoblastoid cell 9, it is a 36 kDa cytosolic protein which adopts a highly conserved 7-bladed β-propeller structure 10, significant homology to the G protein β-subunit 11, and was originally identified as an intracellular protein receptor for protein kinase C. As a member of the tryptophan-aspartate (WD) repeat protein family, RACK1 serves as a scaffold protein for many kinases and receptors, and thus plays a pivotal role in shuttling proteins in intercellular space, anchoring proteins at particular locations, participating in transcriptional/translational events, and stabilizing protein activity. Therein, RACK1 acts as a mediator in multiple signaling pathways, which interconnects distinct signaling pathways to control essential cellular processes, i.e. cell growth, proliferation, migration, adhesion, differentiation, signal transduction, and immune responses 12, 13. It has been reported that RACK1 plays as an oncogene in various tumor types , such as prostate cancer 14, ovarian cancer 15, esophageal squamous cell carcinoma 16, glioma 17, nasopharyngeal carcinoma 18, oral squamous-cell carcinoma (OSCC) 19, breast cancer 20, esophageal carcinoma 21, acute promyelocytic leukemia 22, myeloma 23, colorectal carcinoma 24, 25, non-small cell lung cancer 26, 27, hepatocellular carcinoma (HCC) 28, 29, and etc.

However, the role of RACK1 in melanoma has not yet been reported. The present work demonstrates that RACK1 is highly expressed in melanoma tissues and its expression level is well correlated with the clinical progression of melanoma. Moreover, depletion of endogenous RACK1 lead to decreased proliferation, migration and increased apoptosis of melanoma cells. Thus, our results indicate that RACK1 contributes to the tumorigenesis and progression of melanoma.

## Materials and Methods

### Clinical patient samples

The tissue microarray was obtained from Xi'an Alenabio.com (China), including 67 cases of malignant melanoma tissues, 23 cases of normal skin tissues and 17 cases of benign tissues. 21 cases of fresh human acral melanoma tumor tissues and peripheral non-tumor skin tissues were collected from Affiliated Hospital of Nantong University (Nantong, Jiangsu, China). The study was approved by the Ethics Committee of Affiliated Hospital of Nantong University.

### Cell culture and transfection

Human melanoma cell line A375 and keratinocyte cell line HaCaT were cultured in Dulbecco's modified Eagle's medium (DMEM), and melanoma cell line A875 was cultured in Roswell Park Memorial Institute (RPMI) 1640 medium separately, both containing 10% fetal bovine serum (FBS) (Thermo Fisher Scientific, USA). Cells were maintained at 37 °C with 5% CO_2_. RACK1 targeted siRNA (sense, 5'-CUCUGGAUCUCGAGAUAAAdTdT-3', antisense, 5'-UUUAUCUCGAGAUCCAGAGdTdT-3') was designed and synthesized to knock down the endogenous RACK1 expression in melanoma cells; a non-human homology siRNA sequence was used as negative control (sense, 5'-UUCUCCGAACGUGUCACGUdTdT-3', antisense, 5'-ACGUGACACGUUCGGAGAAdTdT-3'). siRNA was transfected into cells using Lipofectamine® 2000 transfection reagent (Thermo Fisher Scientific, USA) according to the manufacturer's instruction. Both siRNA and its negative control were obtained from Biomics Biotechnologies Co., Ltd. (China).

### Immunohistochemical staining

The tissue microarray sections were determined by immunohistochemical staining using Envision Plus/Horseradish Peroxidase system (DAKO, USA). For immunohistochemical staining, sections were exposed to the primary antibody (1:200 dilution) at 4°C overnight, the antibodies against human RACK1 was obtained from Abcam. After being washed in phosphate-buffered saline (PBS), the sections were incubated with Envision Plus secondary antibody for 30 min, followed by diaminobenzidine solution for 5 min and counterstained with hematoxylin. To eliminate the interference of the brown pigment in melanomas, a negative control omitted primary antibody was applied. The RACK1 staining intensity was evaluated and scored by two pathologists independently. Staining intensity was scored as 0 (negative), 1 (weakly positive), 2 (moderately positive) and 3 (strongly positive). The percentage of positive cells was scored as 0 (<5%), 1 (5-25%), 2 (26-50%), 3 (51-75%) and 4 (>75%). The immunoreactive score (IS) results were evaluated using Remmele-score system (staining intensity score×positive cell percentage score), RACK1 expression was defined as: “-” (score of 0), “+” (score of 1-3), “++” (score of 4-6), “+++” (score of 7-12).

### Real-time quantitative PCR (RT-qPCR)

The cells were collected 48 h after the treatment indicated above; total RNA of cells was isolated using TRIzol^®^ reagent (Thermo Fisher Scientific, USA). The expression of RACK1 mRNA was quantified by RT-qPCR using the One-Step RT-qPCR kit (Thermo Fisher Scientific, USA) according to the manufacturer's manual. Results were analyzed by 2^-ΔΔCt^ method 30, and β-actin was used as an internal control. The primer sequences were as follows: RACK1 forward: 5'-AGATAAGACCATCATCAT-3'; RACK1 reverse: 5'-AGATAACCACATCACTAA-3'; β-actin forward: 5'-TTGCCGACAGGATGCAGAAGGA-3'; β-actin reverse: 5'-AGGTGGACAGCGAGGCCAGGAT-3'.

### Western blot analysis

Western blot was performed according to the standard procedures. Briefly, tissues and cell lysates were extracted using RIPA buffer (Promega, USA), the protein concentrations were determined by a BCA™ Protein Assay Kit (Pierce, USA). Proteins were separated by 10% sodium dodecyl sulfate-polyacrylamide gel electrophoresis (SDS-PAGE) and electro-transferred onto PVDF membranes (Merk-Millipore, USA). The membranes were first incubated with indicated primary antibodies overnight (4 °C), and then followed by HRP-conjugated secondary antibodies for 2 h at room temperature. Blots were detected with ECL Western Blotting Substrate (Promega, USA) and quantized by Image J software (NIH, USA), β-actin was used as an internal control. The following antibodies were included: a rabbit anti-human RACK1 antibody (Abcam, USA, 1:500 dilution), a mouse anti-human β-actin antibody (Abcam, USA, 1:1,000 dilution), a horseradish peroxidase (HRP)-conjugated IgG (Abcam, USA, 1:2,000 dilution).

### Immunocytochemistry staining

Briefly, 2×10^5^ cells were plated into 24-well plates with a round slide in each well and cultured at 37 °C with 5% CO_2_ overnight. After treated for 48 h as described above, the cells were fixed with 4% paraformaldehyde for 30 min at 4 °C, rinsed with PBS, and incubated with 0.5% Triton X-100 for 10 min at room temperature, incubated with the block solution for 30 min at 4 °C, incubated with a rabbit anti-human RACK1 antibody (1:50 dilution) (Abcam, USA) at 4 °C overnight, rinsed with PBS, incubated with IgG-TRITC (1:1,000 dilution) (Abcam, USA) for 2 h at room temperature, rinsed with PBS again and followed by Hoechst 33258 staining (Sigma-Aldrich, USA) for 10 min. Finally, the cells were mounted and observed under an immunofluorescence microscopy protect from light.

### Cell proliferation assay

Cell proliferation was detected with MTT assay. In brief, cells were treated differently as indicated above. After been treated for 24 h, 48 h and 72 h, the cells were incubated with 10 µl MTT (Promega, USA) at 37 °C for 4 h protected from light, followed by 150 µl DMSO incubation at 37 °C for 10 min. Finally, the fluorescence intensity of each well was measured using a microplate reader (Bio-Rad, USA) at spectrometric absorbance of 490 nm.

### Wound-healing assay

The migration of cells was assessed by wound-healing assay. In brief, 1×10^5^ cells were plated onto 6-well plates each well and treated as indicated above. 24 h after treatment, a wound was created by scraping the cell monolayer with 1 ml a pipette tip manually and the medium were replaced with fresh DMEM containing 1% FBS. At 0, 24, or 48 h, cells were washed with PBS, and images were captured under the microscope. Cell migration was estimated by wound healing rate (%) = [1-(wound area at Tt/wound area at T0)]×100.

### Transwell assay

Migration assay was performed using Transwell cell culture systems (Corning, USA). Briefly, 2×10^5^ cells per well were plated onto 24-well plates and cultured for 24 h, after treated for 48 h as described above, cells were suspended in DMEM at the density of 1×10^6^ cells/ml and added to the upper chamber (100 μl/each) while 600 μl medium (conditioned medium: DMEM containing 10% FBS=1:1) was added into the lower chamber, the conditioned medium was the medium from normal cell supernatant cultured for 24 h. After being incubated at 37 °C for 24 h, the cells on the top membrane surface of upper chamber were removed and the infiltrating cells on the bottom surface were fixed in 10% formaldehyde for 30 s, stained with 5% crystal violet for 30 min and observed under a microscope. Cell numbers were counted from five random visions.

### Matrigel-based transwell assay

Cell invasion abilities were detected by matrigel-based transwell assay. Briefly, 2×10^5^ cells per well were plated onto 24-well plates and cultured for 24 h, after treated for 48 h as described above, cells were suspended in DMEM at the density of 1×10^6^ cells/ml. Before treatments, 100 μl matrigel (BD Biosciences, USA) (5 mg/ml) was added into upper chamber and incubated with 300 μl DMEM for 15 min at room temperature. Cells were suspended in DMEM at the density of 1×10^6^ cells/ml and added to the upper chamber (100 μl/each) while 600 μl medium was added into the lower chamber. After incubation at 37 °C for 24 h, the cells on the top membrane surface of upper chamber were carefully removed and cells on the bottom surface were fixed in 10% formaldehyde for 30 s, stained with 5% crystal violet for 30 min and observed under a microscope. Infiltrating cell numbers were counted from five random visions.

### Cell apoptosis assay

Cell apoptotic rate was detected by Annexin V-FITC/Propidium Iodide (PI) followed by flow cytometry (FCM). In brief, 3×10^5^ cells were plated onto 6-well plates per well and incubated for 24 h. Cells were collected after being treated for 48 h, centrifuged at 1,000 rpm for 5 min, washed with PBS, and then suspended in 195 μl 1×Annexin V-FITC binding buffer and 5 μl Annexin V-FITC (Sigma-Aldrich, USA). After 10 min incubation at room temperature protected from light, the cells were centrifuged at 1,000 rpm for 5 min, re-suspended in 190 μl 1×Annexin V-FITC binding buffer, and then incubated with 10 μl PI protected from light. Finally, cells were analyzed using Flow Cytometer (BD Biosciences, USA).

### Statistical analysis

Statistical analyses were performed using SPSS20.0 software. All data was shown as mean ± SD. Paired t-test was used to analyze the difference of RACK1 expression between paired tissues. Significance between multiple groups was evaluated by one-way analysis of variance (ANOVA) followed by a Dunnett post hoc test. For immunohistochemical staining, Kruskal Wallis test was used to analyze the difference of RACK1 expression among three different tissues, Kruskal-Wallis one-way ANOVA followed by all pairwise method was employed to further explore the difference of RACK1 expression between groups. Kendall correlation and Spearman's rank correlation were used to analyze the correlation between RACK1 expression and clinicopathological factors. *P* values are based on a two-tailed statistical analysis. Statistical significance was determined at the level of *P*< 0.05. All assays were performed in triplicate times independently.

## Results

### RACK1 was highly expressed in cell lines and melanoma patients

**Figure 1:**
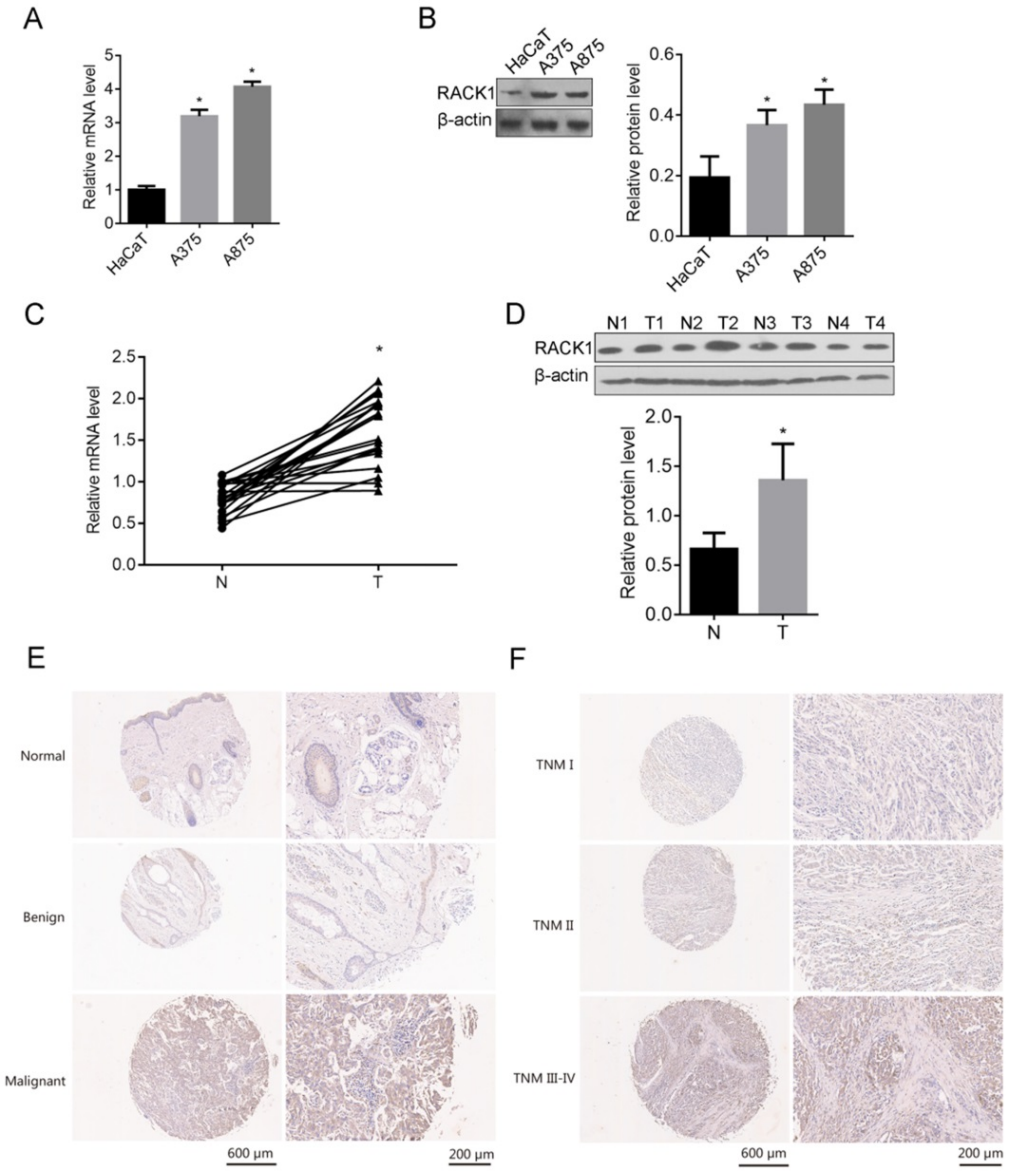
RACK1 expression in melanoma cell lines and tissues from melanoma patients. (A-B) RACK1 mRNA and protein levels in A375, A875 and HaCaT cells were detected by RT-qPCR and Western blot respectively, *P<0.05, compared with HaCaT cells; (C-D) RACK1 mRNA and protein levels in melanoma and paired normal tissues were detected by RT-qPCR and Western blot, *P<0.05, compared with normal tissues; N: non-tumor skin tissues, and T: tumor tissues; (E-F) Immumohistochemical staining of RACK1 expressions in normal, benign and malignant melanoma tissues, and in different stages of melanoma samples (I, II and III-IV), the scale bar indicates 600 μm (left) and 200 μm (right).

To understand whether RACK1 was related to the tumorigenesis of melanoma, We first examined the expression of RACK1 in melanoma cell lines (A375 and A875) and the normal skin cell line HaCaT, both mRNA and protein levels of RACK1 in A375 and A875 cells were higher than that in HaCaT (*P*<0.05) (Fig. 1A and B). The mRNA and protein levels of RACK1 were also checked in tumor tissues from patients with melanoma and the adjacent non-tumor skin tissues, RT-qPCR and Western blot showed that both mRNA and protein levels of RACK1 was higher in melanoma tissues compared with their peripheral non-tumor skin tissues (*P*<0.05) (Fig. 1C and D).

### RACK1 expression was correlated with clinical characteristics in melanoma patients

The expression of RACK1 was explored by immunohistochemical staining in total of 107 tissue samples which including 67 cases of malignant melanoma tissues, 23 cases of normal skin tissues and 17 cases of benign tissues. RACK1 was significantly high expressed in malignant melanoma tissues, compared with that in normal skin tissues and benign tissues (Table 1, Fig. 1E-F). More specifically, 62.69% (42/67) of the melanoma tissues showed strong or moderate staining (+++/++) and 37.31% (25/67) showed weak staining (-/+) of RACK-1, while most of control normal skin and benign tissues showed weak staining (-/+), 91.30% (21/23) and 100% (17/17), respectively. Moreover, RACK1 expression was strongly related with the TNM stage of melanoma (*P*=0.009) (Fig. 1F and Table 2). These results confirmed that RACK is upregulated in melanoma tissues and suggested that RACK1 might contribute to the tumorigenesis and progression of melanoma.

### RACK1 expression was downregulated by siRNA in melanoma cells

**Figure 2:**
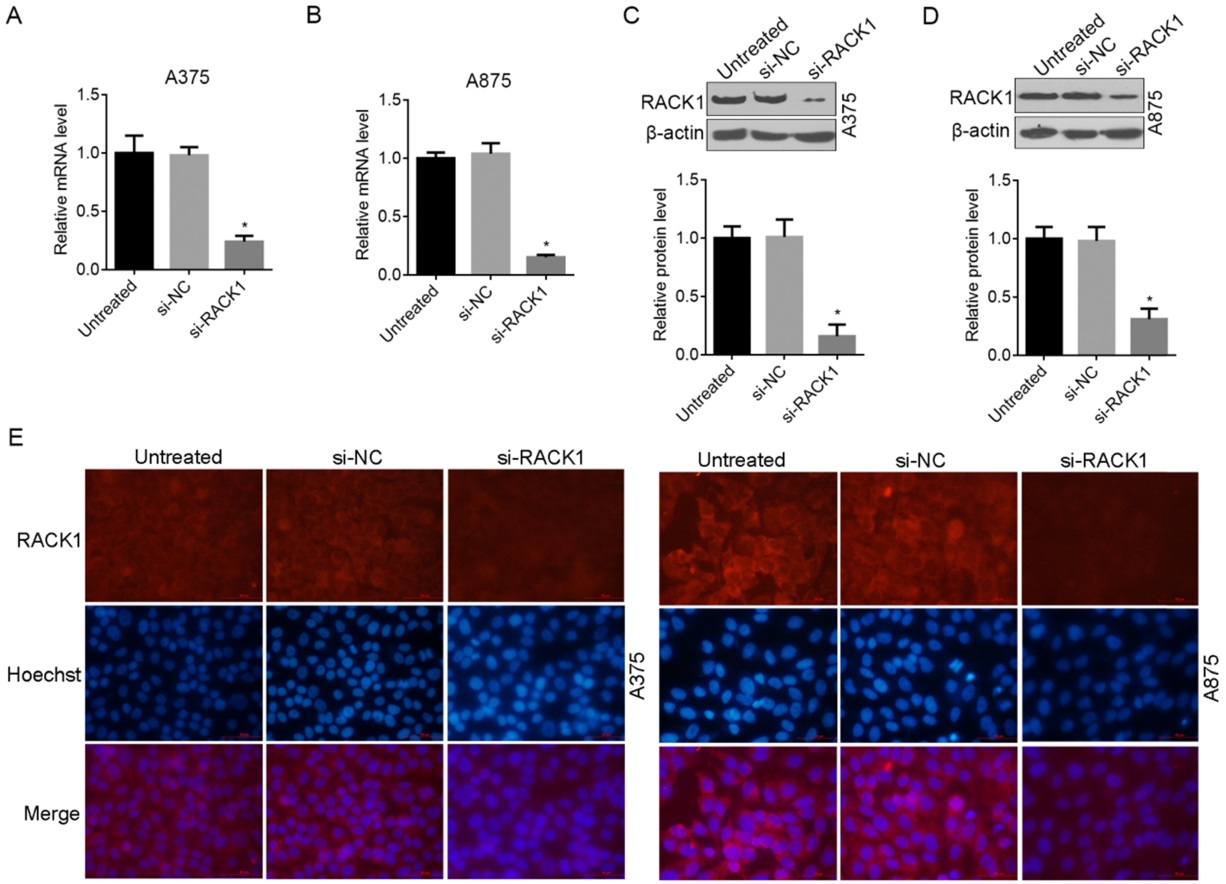
RNAi-induced RACK1 knockdown in melanoma cells. (A-B) RACK1 mRNA levels inhibited by siRNA in A375 and A875 cells was determined by RT-qPCR; (C-D) RACK1 protein levels inhibited by siRNA in A375 and A875 cells was determined by Western blot. *P<0.05, indicated a significant difference from the value of si-NC or untreated cells; (E-F). Knockdown efficiency of RACK1 protein in A375 and A875 cells was determined by fluorescence microscopy, red staining indicated RACK1 expression, cell nucleus was stained with Hoechst33258, the scale bar indicates 50 μm.

To further examine the role of RACK1 in melanoma cells, we designed siRNA (si-RACK1) to downregulate endogenous RACK1 levels in A375 and A875 cells. The interference and knockdown efficiency was confirmed by RT-qPCR (Fig. 2A and B), Western blot analysis (Fig. 2C and D) and fluorescence microscopy (Fig. 2E). All the above data proved that compared with si-NC treated or untreated cells, RACK1 expression was significantly inhibited by si-RACK1 in both A375 and A875 cells (Fig. 2).

### The growth of melanoma cells was inhibited by RACK1 downregulation

**Figure 3:**
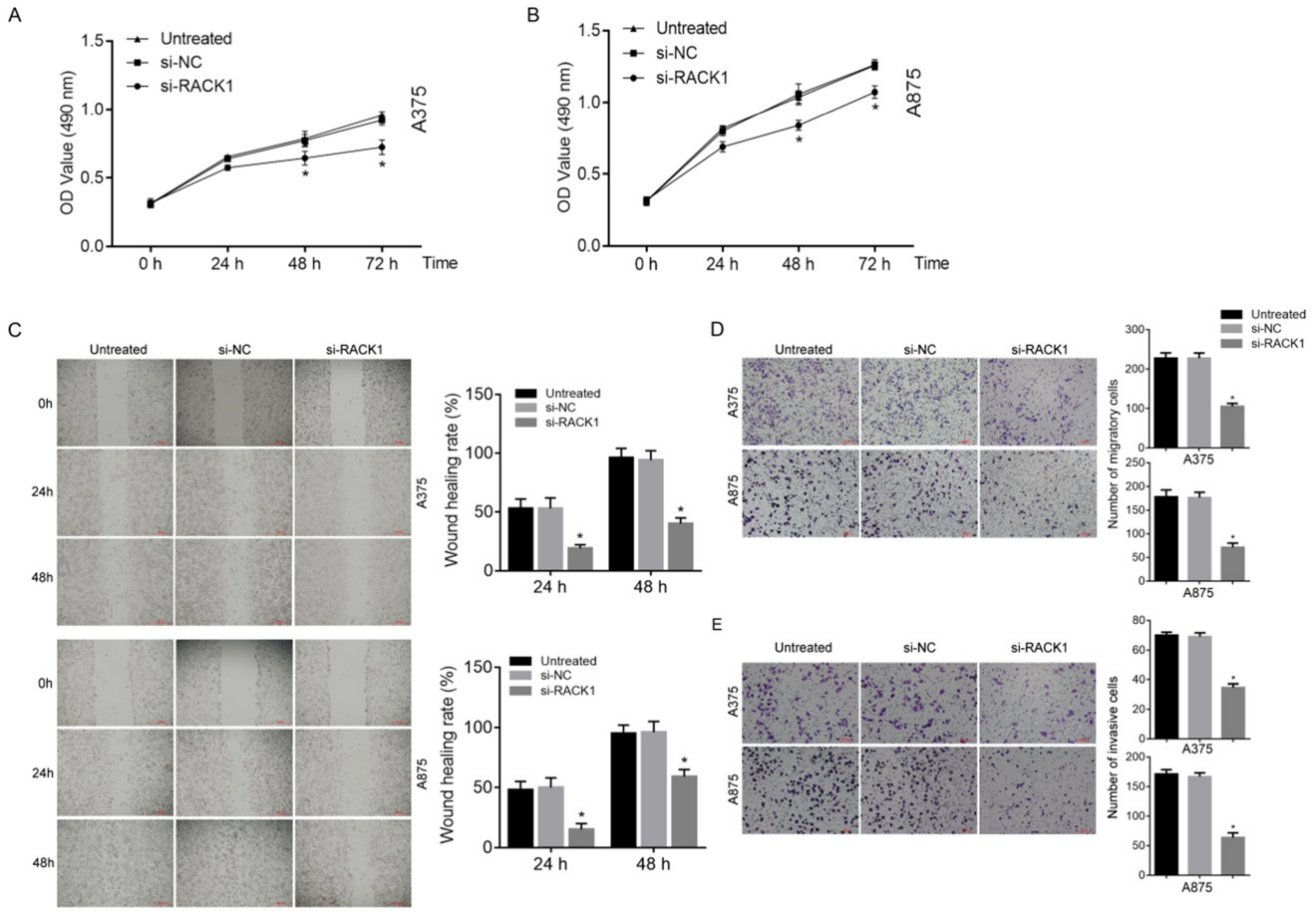
RACK1 siRNA inhibited the proliferation, migration and invasion of melanoma cells. Untreated, si-NC and si-RACK1 melanoma cells were applied to investigate the effect of RACK1 RNAi on the function of melanoma cells. (A-B) The proliferation of A375 and A875 cells was detected by MTT assay. (C) The migration of A375 and A875 cells was detected by wound-healing assay, the scale bar indicates 500 μm. (D) The migration of A375 and A875 cells was detected by transwell assay, the scale bar indicates 100 μm. (E) The invasion of A375 and A875 cells was detected by matrigel-transwell assay, the scale bar indicates 100 μm. All *P<0.05, compared with si-NC or untreated cells.

MTT assay was utilized in order to investigate the inhibitory effects of RACK1 downregulation on the growth of melanoma cells. The result showed that compared with si-NC treated or untreated cells, si-RACK1 clearly inhibited the growth of A375 and A875 cells at 48 h and 72 h (*P*<0.05) (Fig. 3A and B).

### The migration and invasion of melanoma cells was inhibited by RACK1 downregulation

Both wound-healing and transwell assay were used to observe the effect of RACK1 downregulation on melanoma cell migration. Matrigel-based transwell assay was utilized to evaluate the influence of RACK1 depletion on melanoma cell invasion. Compared with that of si-NC treated or untreated cells, si-RACK1 treatment significantly decreased both migration (*P*<0.05, Fig. 3C and D) and invasion (*P*<0.05, Fig. 3E) of A375 and A875 cells.

### The apoptosis of melanoma cells was induced by RACK1 downregulation

**Figure 4:**
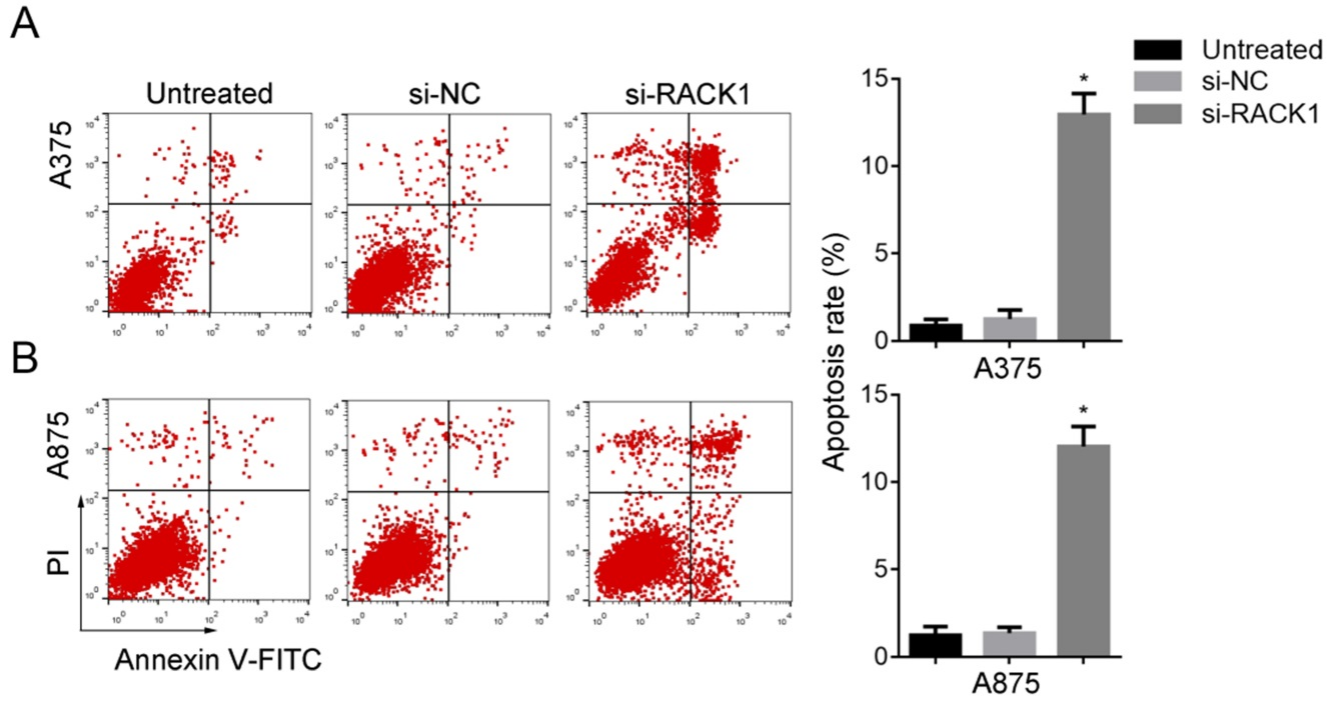
RACK1 siRNA promoted the apoptosis of melanoma cells. Untreated, si-NC and si-RACK1 melanoma cells were fixed and stained with PI, the apoptotic rates were counted by FCM analysis. (A) A375 and (B) A875 cell apoptosis. *P<0.05, compared with si-NC or untreated cells.

Annexin-V-FITC/PI followed by FCM was performed to evaluate the effect of RACK1 downregulation on melanoma cell apoptosis. Compared with that of si-NC treated or untreated cells, si-RACK1 treatment significantly increased apoptosis of A375 and A875 cells (*P*<0.05) (Fig. 4).

## Discussion

Melanoma is a highly malignant tumor with limited treatment options 1. Despite much research, the mechanisms of the occurrence and development of melanoma remains unclear. Surgery, radiotherapy and chemotherapy have long been the main methods for melanoma treatment, but these methods also have some problems, such as severe trauma, obvious side effects and low patient tolerance 31. With the recent process in treatment, immune therapies and targeted therapies are two main treatment approaches for patients with advanced melanoma, but both still have limitations, and not all patients experience sustained responses 32.

RACK1 was originally indentified as a key anchor for protein kinase C 11, and has been shown to be involved in numerous different biological processes. In recent years, RACK1 has been reported as an oncogene in many human cancers 13. But it is interesting to note that the role of RACK1 in different tumors is not the same and sometimes even the opposite. Cao et al. demonstrated that RACK1 was high expressed in breast cancer and was a prognostic factor that promoted breast carcinoma metastasis by interacting with RhoA and activating the RhoA/Rho kinase pathway 33. RACK1 has also been found highly expressed in human HCC, which contributed to in vitro chemoresistance and in vivo tumor growth and survival of HCC 34. In non-small-cell lung cancer, RACK1 served as an oncogene and silence of RACK1 resulted in inhibition of tumor growth and metastasis through the sonic hedgehog signaling pathway 35. Highly expression of RACK1 was also found in prostate cancer 14, colorectal cancer 24, 25 and nasopharyngeal carcinoma 36. But in gastric cancer and pancreatic ductal adenocarcinoma, things have changed. Deng et al. demonstrated that RACK1 was decreased in gastric cancer patients and associated with tumor infiltration depth and poor differentiation, indicated that RACK1 suppressed gastric tumorigenesis 37. The tumor suppressor role of RACK1 in gastric cancer was also supported by Chen et al, indicated that low expression of RACK1 was correlated with invasion and metastasis phenotype as well as 5-year survival in clinical cases, suggesting RACK1 decrease in gastric cancer links epigenetics to interleukin 8 (IL8) to promote tumor metastasis 38. Additionally, low RACK1 expression was involved in pancreatic cancer growth and metastasis, and significantly correlated with metastasis, invasion of nerves as well as TNM stage; especially 3-year survival rate of pancreatic ductal adenocarcinoma patients with high RACK1 expression was significantly higher than those patients with low ones 39. These studies have elucidated that the function of RACK1 is very complex and varies in different cancers. In this study, we reported that RACK1 was highly expressed in melanoma tissues compared with matched nontumorous skin tissues. The results were also supported by tissue microarray staining data: RACK1 expression in tumor tissues was significantly higher than that in benign lesions and normal skin tissues. Moreover, high levels of RACK1 in melanoma tissues were highly correlated with TNM stage (*P*=0.009), strongly predicted that RACK1 expression was associated with the progression of melanoma.

Evidences indicate that RACK1 participating in multiple cell functions. Silencing of RACK1 resulted in inhibition of tumor growth of non-small-cell lung cancer cells 35, induced cell apoptosis and inhibited cell proliferation of HCC and glioma cells 40, 41; RACK1 has also been found to be engaged in the regulation of cell migration and invasion, two important initial steps in cell metastasis, because it served as a molecular bridge linking the integrin effector focal adhesion kinase to the recruitment of a cAMP-degrading phosphodiesterase in the “direction-sensing” pathway 42, such phenomenon was confirmed in various tumor types, such as prostate cancer cells, epithelial ovarian cancer cells, esophageal squamous cell carcinoma cells, glioma cells, colorectal cancer cells, OSCC cells and nasopharyngeal carcinoma cells 14-17, 24, 41, 43, 44.

To understand what cellular processes are modulated by RACK1 in melanoma, we investigated the effect of RACK1 downregulation on the functions of A375 and A875 cells. In the present study, RACK1 knockdown resulted in inhibition of cell proliferation and promotion of cell apoptosis of A375 and A875 cells; *in vitro* study also showed that the migration and invasion of melanoma cells were inhibited. It is worth noting that, the significantly correlation of RACK1 overexpression with metastasis of melanoma patients has not been found in our study, which may be due to tumor heterogeneity and the limited samples used here, it is necessary to further study in a larger sample size.

In conclusion, the present work suggests that RACK1 may be a factor affecting the prognosis of melanoma, and the strategy of RNAi-mediated RACK1 silencing may be an effective method for melanoma treatment.

## Figures and Tables

**Table 1:** Difference of RACK1 expression in malignant melanoma and normal or benign skin tissues evaluated by immunoreactive score grade (ISG).

Type	*n*	RACK1 expression, *n*				Kruskal Wallis Test			Kruskal-Wallis one-way ANOVA (all pairwise)
		(-)	(+)	(++)	(+++)	Mean Rank	*x^2^*	*P* value	*P* value
Normal	23	6	15	2	0	31.30	35.354		0.00**
Benign	17	1	16	0	0	34.15		0.00*	0.00**
Malignant melanoma	67	1	24	24	18	66.83			
Total	107	8	55	26	18				

ISG: 0 (-), 1~3 (+), 4~6 (++), 7~12 (+++). *P<0.05; **P<0.05 vs malignant melanoma.

**Table 2:** Correlation between RACK1 expression and clinical characteristics in melanoma patients.

Clinical characteristics	n	RACK1 expression				*P* value
		(-)	(+)	(++)	(+++)	
Gender						
Male	40	1	9	18	12	0.059
Female	27	0	15	6	6	
Age (years)						
<55	38	0	12	14	12	0.193
≥55	29	1	12	10	6	
TNM						
I	5	0	3	2	0	0.009^*^
II	36	1	15	13	7	
III-IV	26	0	6	9	11	
Lymphatic metastasis						
Negative	45	1	18	16	10	0.153
Positive	22	0	6	8	8	

*P<0.05

## Abbreviations

RACK1: receptor for activated C kinase 1
RNAi: RNA interfering
WD: tryptophan-aspartate
OSCC: oral squamous-cell carcinoma
HCC: hepatocellular carcinoma
DMEM: Dulbecco's modified Eagle's medium
RPMI: Roswell Park Memorial Institute
FBS: fetal bovine serum
PBS: phosphate-buffered saline
RT-qPCR: real-time quantitative PCR
SDS-PAGE: sodium dodecyl sulfate-polyacrylamide gel electrophoresis
HRP: horseradish peroxidase
PI: propidium Iodide
FCM: flow cytometry
IL8: interleukin 8
